# Age-Related Differences in Resting-State Functional Connectivity Predict Specific Patterns of Speech Disfluency

**DOI:** 10.1162/NOL.a.245

**Published:** 2026-04-23

**Authors:** Megan S. Nakamura, Haoyun Zhang, Michele T. Diaz

**Affiliations:** The Pennsylvania State University, University Park, PA; Centre for Cognitive and Brain Sciences, Department of Psychology, University of Macau, Taipa, Macau SAR, China

**Keywords:** aging, disfluency, functional magnetic resonance imaging (fMRI), resting-state functional connectivity (RSFC), speech production

## Abstract

Fluent speech production remains largely preserved across adulthood, yet subtle disruptions such as pauses, repetitions, and revisions become more common with age. These disfluencies may reflect underlying cognitive and neural changes that accompany aging, particularly in executive function (EF) and large-scale brain network organization. In this study, we examined whether EF and resting-state functional connectivity (RSFC) independently or jointly explained age-related differences in naturalistic speech disfluencies in an adult lifespan sample (*n* = 252, ages 20–81 years). RSFC was used to assess network segregation within three systems implicated in language and cognitive control: language network, default mode network (DMN), and multiple demand (MD) network. These task-independent connectivity patterns provide insight into how the brain’s functional architecture impacts speech production and its age-related vulnerabilities. Our findings indicate that age was associated with increased rates of specific disfluency subtypes, such as unfilled pauses, repetitions, and revisions, as well as lower EF and lower language, MD, and DMN network segregation. Although increasing age was associated with lower EF, EF performance did not predict disfluencies or mediate their age-related increase. In contrast, higher DMN segregation predicted lower overall disfluencies, repetitions, and revisions. Age moderated the relationship between DMN segregation and repetitions, with a significant association only in younger and middle-aged adults, suggesting weaker brain–behavior relationships at older ages. DMN segregation also partially mediated the relationship between age and revisions. These findings suggest that while EF relates to planning-related disruptions, changes in functional brain organization may more directly contribute to age-related increases in self-monitoring disfluencies.

## INTRODUCTION

Although speech remains a highly effective tool for communication across the lifespan, the process of producing fluent speech becomes increasingly effortful with age. Even in healthy aging, subtle disruptions in speech fluency, i.e., disfluencies, become increasingly common. These disfluencies may offer sensitive behavioral markers of underlying cognitive and neural changes that accompany the aging process. However, the mechanisms driving these changes remain poorly understood. The present study addresses this issue by examining how executive function (EF) and resting-state functional connectivity (RSFC) contribute to age-related differences in naturalistic speech disfluencies.

### Speech Disfluencies Increase with Age

Compared to younger adults, older adults’ speech is often characterized as more disfluent, i.e., having more momentary disruptions—including repetitions, revisions, pauses, and prolongations—which have been posited to reflect the complex demands of planning, retrieving, articulating, and monitoring language in real time (Bóna, 2011; Bortfeld et al., 2001; Kemper et al., 2009; Maclay & Osgood, 1959). Moreover, studies have found a positive relationship between age and disfluencies across a variety of tasks including conversational speech (e.g., Horton et al., 2010), narrative retelling (e.g., Lee et al., 2019; Saling et al., 2012), and picture description (e.g., Castro & James, 2014; Dennis & Hess, 2016). However, it should be noted that these findings are mixed, with some studies reporting minimal or no age differences in disfluency frequency, particularly in structured or highly scaffolded tasks, e.g., picture naming, sentence repetition, etc. (e.g., Wright et al., 2014; see review in Mortensen et al., 2006). Age differences also tend to diminish when overall speech rate is controlled (Kemper et al., 2003), suggesting that some disfluency increases may stem from general slowing or reduced speech quantity rather than changes in language specific processes per se.

Such variability, however, does not undermine the cognitive relevance of increased disfluencies with aging. Rather, it highlights that speech disfluency may reflect a complex interplay between age-related decline and adaptative strategies. For instance, older adults may pause more often to compensate for slower lexical access and retrieval or to plan for longer utterances (Kemper et al., 2010, 2011; Spieler & Griffin, 2006). On the other hand, filled pauses such as “um” and “uh” may serve communicative or pragmatic functions, allowing speakers to manage turn-taking and signal temporary delays in speech planning (Clark & Fox Tree, 2002). In this way, disfluencies may reflect not just breakdowns in communication but also adaptive mechanisms for effectively managing cognitive demands in the face of age-related cognitive decline.

One proposed explanation for age-related increases in speech disfluency is a decline in EF, given that producing fluent speech requires not only accessing and organizing lexical and syntactic information but also continuously monitoring and updating output as speech unfolds in real time. These processes rely on domain-general cognitive resources such as attention, inhibition, and working memory that become more limited with age (Kemper et al., 2001; see also Salthouse, 1996). Supporting this view, studies using dual-task paradigms have found that during higher cognitive loads, older adults exhibit disproportionate increases in disfluency and reduced grammatical complexity compared to younger adults (Kemper et al., 2003), suggesting that fluency may be sensitive to the availability of cognitive resources. However, EF decline is just one of several proposed mechanisms. Other accounts emphasize general slowing (Salthouse, 1996) or weakened connections between phonological and semantic representations (Burke & Shafto, 2004). Collectively, these views underscore the multifaceted nature of age-related changes in speech production.

### Disfluency Subtypes

As previously mentioned, disfluency subtypes may reflect distinct cognitive and linguistic mechanisms related to speech planning, monitoring, and control (e.g., Engelhardt et al., 2013, 2019; Fraundorf & Watson, 2013). In the present study, we focus on five disfluency subtypes: repetitions (e.g., the-the frog), revisions (self-corrections, e.g., the dog … I mean the frog), unfilled pauses (intervals of silence), filled pauses (e.g., *um, uh*), and prolongations (e.g., *Theeee frog went* …) to examine if these subtypes are differentially impacted by age. Unfilled pauses have been posited to signal difficulties in maintaining access to target lexical or syntactic forms during speech planning (Kemper et al., 2010, 2011). By contrast, filled pauses have been proposed to potentially serve compensatory functions. Whereas some instances reflect planning difficulty, filled pauses can also help speakers manage turn-taking or signal temporary processing delays (Clark & Fox Tree, 2002). The mixed pragmatic-cognitive profile of filled pauses makes them uniquely informative for distinguishing communicative strategies from production breakdowns. Additionally, revisions require detection of an error, interruption of output, and initiation of a corrected structure—all of which draw on monitoring. Repetitions are among the most common disfluencies observed in older adults (Engelhardt et al., 2010), and evidence suggests a U-shaped trajectory across the lifespan, with high rates in early childhood, lower rates in young adulthood, and a resurgence in later life (Bortfeld et al., 2001; Yairi & Clifton, 1972). Together, these subtypes underscore that disfluency is not a monolithic indicator of decline but rather a dynamic behavioral signal of how cognitive and linguistic resources are managed during speech production at different stages across the lifespan. Disentangling speech disfluency by categories is therefore crucial for understanding how cognitive aging affects speech, as the prevalence of each subtype may provide a more precise behavioral signature of which processes are compromised or preserved.

### EF and Fluent Speech in Aging

EF is central to understanding the link between age-related changes in speech disfluency and cognition. EF refers to a set of higher-order cognitive processes—including inhibition, attentional control, task switching, monitoring, and updating—that enable goal-directed regulation of thought and behavior (Diamond, 2013; Miyake et al., 2000). Language production generally places substantial demands on EF (Rabaglia & Salthouse, 2011). For instance, inhibition is required to prevent the intrusion of irrelevant lexical or syntactic material, while working memory supports the maintenance and manipulation of unfolding sentence structure. Compromises in these executive control processes can therefore manifest as increases in disfluencies (Engelhardt et al., 2010; Shao et al., 2014).

In parallel, age-related declines in EF are well documented in the cognitive aging literature. Older adults typically exhibit slower responses, increased error rates, and reduced flexibility across tasks measuring inhibition, task switching, and/or working memory capacity (e.g., Fisk & Sharp, 2004; Salthouse, 1996; Salthouse et al., 2003). These declines can significantly impact language production, especially in situations where speech must be produced under pressure or when managing competing lexical or conceptual representations.

Recent evidence suggests that individual differences in EF predict age-related variation in speech fluency. Older adults with poorer EF scores are more likely to produce disfluencies—especially repetitions and revisions—during spontaneous narrative or picture description tasks (Kemper et al., 2001; Spieler & Griffin, 2006). Moreover, when speech planning is constrained by time pressure or increased lexical competition, speakers with limited EF resources are more prone to producing fragmented or disfluent utterances (Engelhardt et al., 2013). These findings reinforce the idea that EF is crucial for coordinating complex linguistic operations, especially under demanding processing contexts.

It is important to note, however, that increased disfluency is not universal among older adults. Those with relatively preserved EF tend to exhibit more fluent and syntactically complex speech, suggesting that EF may protect or compensate for age-related language decline (Kemper et al., 2010).

### Brain Network Reorganization With Age

While EF plays a central role in language production, recent research has highlighted how age-related changes in large-scale brain networks may underlie declines in both EF and speech fluency. RSFC has emerged as a robust tool for examining these changes, revealing how aging alters the coherence and coordination of neural systems in the absence of task demands. Across the adult lifespan, brain networks have been shown to undergo functional dedifferentiation, a broad process in which neural systems progressively lose their distinct boundaries and specialized roles (Li et al., 2001). Although the term “deafferentation” originally referred to reduced category specificity of stimulus-evoked activity (e.g., Li et al., 2001; Park et al., 2012), contemporary network neuroscience has extended this principle to the systems level, where dedifferentiation can be conceptualized as reduced functional segregation of large-scale networks. In this framework, age-related decreases in network segregation reflect weaker within-network cohesion and increased cross-network coupling (e.g., Betzel et al., 2014; Chan et al., 2014; Malagurski et al., 2020).

Segregation therefore serves as a key RSFC metric of the brain’s modular organization, where high segregation reflects efficient architecture, with strongly integrated activity within networks and limited interactions between networks (Chan et al., 2014), and age-related declines in segregation signal diminished functional specificity (e.g., Goh, 2011; Malagurski et al., 2020; Rieck et al., 2021).

These effects are especially pronounced in associative networks, including the default mode network (DMN) and multiple demand (MD) network. The DMN, typically active during rest, has been associated with internally directed cognition (e.g., autobiographical memory, mind wandering, and introspection) and shows marked decreases in within-network connectivity with increased age (e.g., Andrews-Hanna, 2012). This increased cross-talk may impair the brain’s ability to shift flexibly between internally and externally focused tasks. Similarly, the MD network, which supports cognitive control, working memory, and interference resolution, exhibits reduced segregation in older adults (Fedorenko, Duncan, & Kanwisher, 2013; Grady et al., 2010; Hoffman & Morcom, 2018), potentially undermining the regulation of goal-directed behavior.

Growing evidence links reduced segregation to poorer behavioral performance, including declines in global cognition and language outcomes (Zhang et al., 2021). These organizational changes have important implications for speech production, which requires coordination between language-specialized perisylvian regions and domain-general networks supporting monitoring, control, and planning (Hertrich et al., 2020; Mineroff et al., 2018; Seghier & Price, 2012). Thus, age-related disruptions in network segregation, particularly within and between the language, DMN, and MD networks may contribute to increased speech production disfluency observed in older adults.

### Network Segregation and Language Production in Aging

Building on evidence that age-related declines in network segregation mark broader cognitive aging, a growing body of RSFC research has begun to investigate how these changes affect language production specifically. Much of this work has focused on associative networks, e.g., the DMN and MD, which reliably show that age-related decreases in segregation predict poorer cognitive outcomes (Damoiseaux, 2017; Onoda et al., 2012; Pedersen et al., 2021).

However, unlike these domain-general systems, the language network appears to show greater resilience in later life (Campbell et al., 2016; Samu et al., 2017). For example, Campbell et al. (2016) reports robust maintenance of frontotemporal syntax network connectivity across the lifespan, with no reduction in within-network specialization despite age-related structural atrophy. Samu et al. (2017) similarly reports that only cognitive domains exhibit behavioral decline (e.g., fluid intelligence, object naming), whereas syntactic comprehension and its associated neural systems remained stable.

Consistent with this pattern, recent RSFC findings indicate that segregation within the language network declines more slowly than in domain-general systems, even as whole-brain segregation decreases (Zhang & Diaz, 2023). In parallel, age differences in the DMN’s subsystems highlight that DMN integrity is more vulnerable to aging than language-specific networks (Campbell et al., 2013). Together, these results suggest that age-related speech difficulties may not reflect degradation of the language network itself but rather altered interactions between language regions and domain-general systems.

This perspective is supported by work demonstrating functional dissociations among the language, DMN, and MD networks (Mineroff et al., 2018) and by task-based evidence showing that successful language production often recruits frontoparietal and medial regions involved in conceptual planning, monitoring, and cognitive control (e.g., Fedorenko & Thompson-Schill, 2014). Despite these links, relatively few studies have examined how intrinsic functional architecture relates to spontaneous speech in older adults.

Moreover, most RSFC–language studies focus on global fluency metrics (e.g., word count, mean length of utterance) or task-based paradigms under constrained conditions (Engelhardt & Markostamou, 2025). Yet, disfluencies, particularly when categorized into subtypes such as repetitions, revisions, unfilled pauses, and filled pauses, may reflect distinct underlying cognitive disruptions at the neural-network level. Therefore, examining how aging affects the coordination of language and domain-general systems, specifically network segregation within the language, DMN, and MD networks, can shed light on the neural mechanisms that possibly contribute to disfluency in older adults.

### The Current Study

The present study investigates how age-related differences in network organization contribute to variation in spontaneous speech fluency. Building on prior work linking EF and functional connectivity to language production, we examine whether network segregation within the language, DMN, and MD networks predicts disfluency subtypes in naturalistic speech. We focus on network segregation—a measure of how well a network maintains functional boundaries from other systems—as it has been shown to be a sensitive indicator of cognitive aging and a potential mechanism underlying age-related declines in EF. Given evidence that the DMN and MD networks are particularly vulnerable to aging, whereas the language network shows greater resilience, we test whether lower segregation in the DMN and MD networks, but not the more age-resilient language network, is associated with greater disfluency.

Disfluencies are examined both as a total proportion and broken down into subtypes (repetitions, revisions, unfilled pauses, and filled pauses) to capture distinct cognitive mechanisms. Based on prior findings, we predicted that repetitions and revisions in particular, which involve conflict resolution and self-monitoring, would show the strongest associations with aging and network-level disruption.

We also evaluate whether EF, measured here using the color-word Stroop task, mediates the relationship between age and disfluency. Although Stroop interference captures primarily inhibitory control, it is widely used as a behavioral proxy for EF in cognitive research (Friedman & Miyake, 2004; Miyake et al., 2000). Thus, we treat Stroop performance as a focused but established behavioral proxy for EF. We predict that age-related increases in disfluency types would be partially explained by declines in EF. Finally, we test whether network segregation mediates age-related increases in disfluency, offering a complementary neural pathway through which aging may affect speech production. By distinguishing among disfluency subtypes and integrating both EF and network-level organization, this study aims to identify specific cognitive and neural mechanisms contributing to different types of speech disfluency in aging.

## METHODS

### Participant Demographics

Data were collected across three separate but related adult lifespan sample language production studies (Diaz et al., 2021, 2022). In Study 1 (*n* = 91), participants completed a narrative retelling task. In Study 2 (*n* = 90) and Study 3 (*n* = 91), participants completed two naturalistic speech tasks (a narrative retelling and an open-ended prompt; see details in the Task Descriptions section below). A total of 272 adults (ages 20–81 years) participated in these studies. All participants were community-dwelling, right-handed, native English speakers who were not fluent in a second language. All participants had normal or corrected-to-normal vision and reported no history of neurological, psychological, or major medical conditions (Christensen et al., 1992). Participants were excluded if they failed cognitive screening (Mini-Mental State Exam [MMSE] < 24 or MoCA [Montreal Cognitive Assessment] < 26), were missing key variables (age, Stroop, or RSFC, or disfluency measures), or were identified as outliers using Cook’s distance (threshold = 4/*n*). Four participants were removed from the analysis because of missing data points, i.e., did not complete the story elicitation task, and an additional 15 participants were removed due to having outlier data points on the behavioral measures of speech disfluency (see Data Analysis for details), leaving 253 participants’ data in the final analyses (ages 20–81 years, mean age = 46.83 years, *SD* = 17.1 years, 156 female).

### Task Descriptions

All participants first completed a behavioral testing session with screening measures and a battery of psychometric and neuropsychological tests to assess basic cognitive functions such as speed, EF, memory, and language. The screening measures included the MMSE (Folstein et al., 1975) or the MoCA (Nasreddine et al., 2005) to screen for mild cognitive impairment or dementia; one study administered the MMSE, while the other two used the MoCA. Additionally, the Geriatric Depression Scale Short Form was used to screen for symptoms of depression (Guerin et al., 2018; Sheikh & Yesavage, 1986).

The cognitive tasks included measures of processing speed (simple and choice reaction time, and digit–symbol substitution), vocabulary knowledge (Wechsler Adult Intelligence Scale-III vocabulary), working memory (forward and backward digit-span), verbal working memory (reading span task), and episodic memory (California Verbal Learning Test). To measure EF, participants completed a color-word Stroop task, which required participants to name the ink color of a printed word while ignoring the word’s meaning (e.g., saying “blue” when the word “red” is printed in blue ink). This task taxes inhibitory control by requiring participants to suppress the automatic tendency to read the word in favor of identifying its ink color. The task included both congruent trials (e.g., red in red ink) and incongruent trials (e.g., red in blue ink).

Language assessments included the author recognition test and a comparative reading habits questionnaire to assess exposure to print (Acheson et al., 2008). Participants also completed two verbal fluency tasks, i.e., a phonemic and semantic version. For the phonemic version, participants were instructed to produce as many words as possible within 1 min that start with a letter (F, A, S), and for the semantic version, participants were asked to produce as many words as possible that belong to a certain category (e.g., animals, supermarkets).

Speech production was elicited using two types of open-ended speech tasks that varied across data sets. For two of the projects, participants completed both a narrative retelling of the wordless picture book *Frog, Where Are You?* (Mayer, 1969), where they were asked to describe the story as if the experimenter had never seen it before, as well as respond to an open-ended prompt: “What do you like about where you live?” For one of the projects, participants completed only the narrative-retelling task. All speech tasks were audio recorded.

In what follows, we focus specifically on the Stroop task as the key measure of EF and speech elicitation tasks to reflect language production. All other cognitive and language assessments are included to characterize the broader cognitive profile of participants but were not analyzed further. Descriptive statistics for all measures are reported in Table 1. All participants provided written informed consent and received monetary compensation ($15–30/hr) for their participation. All procedures were approved by the institutional review board at The Pennsylvania State University.

**Table 1.** Cognitive and linguistic baseline characteristics of participants

Demographic information	*M* (*SD*)	Range	Age regression
*N*	252		
Age (years)	46.83 (17.1)	20–81	
Gender (M/F)	96/156		
Education (years)	17.21 (2.9)	6–28	0.01
MMSE (score out of 30)	28.98 (1)	26–30	−0.01
MoCA (score out of 30)	28.67 (1.23)	26–30	−0.02
Depression (GDS) (score out of 15)	0.90	0–6	−2.43*
Cognitive assessments			
Simple RT (box, ms)	291.81 (65.68)	213.4–957.55	1.03*
Choice RT (arrow, ms)	334.27 (74.47)	248.18–781.87	2.09*
WAIS vocabulary (score out of 66)	54.82 (5.68)	32–69	0.02
Digit symbol RT (ms)	1536.32 (371.15)	873.53–3523.12	14.47*
Digit span forward (score out of 16)	11.25 (2.25)	4–16	−0.01*
Digit span backward (score out of 16)	7.32 (2.09)	3–14	−0.02*
Verbal WM (score out of 1)	0.75 (0.15)	0.1–0.99	−0.023*
Immediate recall (score out of 16)	10.88 (2.46)	3–16	−0.03*
Delayed recall (score out of 16)	9.32 (2.87)	2–16	−0.03*
Stroop effect RT (ms)	60.45 (77.9)	−63.68–419.95	1.72*
Author recognition test	24.05 (13.92)	3–64	0.39*
Comparative reading score	25.45 (4.69)	11–35	0.034
Language Production Measures			
VF phonemic (F, A, S)	43.27 (12.46)	15–84	−0.05*
VF category (animal and supermarket)	48.6 (9.41)	27–76	−0.14*
Picture naming (accuracy)	0.72 (0.08)	0.46–0.83	−0.00

*Note*. Age regression shows unstandardized slopes from separate linear regressions predicting each measure from age (in years). Positive values indicate higher scores with increasing age; negative values indicate lower scores with increasing age. Asterisks indicate *p* < 0.05.

### Behavioral Measures

#### Speech disfluency coding

All speech tasks were audio recorded and transcribed using the Computerized Language Analysis software suite (MacWhinney, 2000). Transcriptions adhered to CHAT (Codes for the Human Analysis of Transcripts) conventions, a standardized system of annotating spoken language that captures, e.g., utterance boundaries, disfluencies, speaker turns, etc. (see details in MacWhinney, 2000). Disfluencies were manually coded by a trained coder and categorized into five subtypes: filled pauses (e.g., *uh, um*), unfilled pauses (silent intervals exceeding 1 s), repetitions (e.g., *he he he is going*), revisions (e.g., *he went—I mean, he walked*), and prolongations (e.g., segmental lengthening of a phoneme within a word, such as /s/ in *ssssoup* or /ɔ/ in *loooong*).

#### Stroop coding

EF performance was operationalized as the Stroop interference effect, which was calculated as the difference in mean reaction time (in milliseconds) between incongruent and congruent trials, with larger values indicating greater difficulty inhibiting the prepotent response (see details in MacLeod, 1991; Scarpina & Tagini, 2017). These scores were used as a predictor in regression models examining whether EF performance was associated with speech disfluency outcomes and as a mediator in mediation models testing whether age-related changes in EF partially explained the link between age and disfluency rates. Note that because Stroop interference specifically indexes inhibitory control, we use it here as a focused measure of this subcomponent rather than a global EF metric. In what follows, however, we use the term “EF” to refer to Stroop performance as an established proxy for executive functioning.

### MRI Acquisition and Preprocessing

#### MRI data acquisition

All imaging data were acquired on a 3T Siemens Prisma Fit scanner using a 64-channel head coil. Localizer images were collected to define a volume for data collection, higher-order shimming, and alignment to the anterior commissure and posterior commissure (AC–PC). T1-weighted anatomical images were then collected using a magnetization-prepared rapid acquisition gradient echo sequence (repetition time [TR] = 2,300 ms; echo time [TE] = 2.28 ms; inversion time = 900 ms; flip angle = 8°; echo spacing = 7 ms; acceleration factor = 2; field of view [FOV] = 256 mm^2^; voxel size = 1 × 1 × 1 mm; 160 contiguous slices). RSFC images were collected immediately after the T1 images, and during the scan, participants were instructed to relax with their eyes open while they viewed a fixation cross. Blood oxygen level-dependent (BOLD) resting-state data were acquired using an echoplanar imaging sequence (TR = 2,000 ms; TE = 25.0 ms; flip angle = 90°; echo spacing = 0.49 ms; FOV = 240 mm^2^; voxel size = 3 × 3 × 4 mm; 33 contiguous slices, parallel to the AC–PC; phase encoding = anterior to posterior, fat saturation = on; slice acquisition = sequential, descending; volumes = 180; run duration = ~6 min). Two additional volumes were acquired and deleted at the start of the scan to reach steady state equilibrium. In addition, a field map was collected using a double-echo, spoiled gradient echo sequence (TR = 446 ms; TEs = 4.92 and 7.38 ms; flip angle = 60; FOV = 240 mm^2^; voxel size = 3 × 3 × 4 mm; 33 contiguous slices; phase encoding = anterior to posterior, fat saturation = off; duration = 1:12 min) that generated two magnitude images and one phase image to correct for field inhomogeneity.

#### Preprocessing pipeline

Anatomical and functional data were visually inspected for artifacts and signal dropout. Functional data quality was more formally assessed using the fBIRN QA tool, measuring the number of potentially clipped voxels, mean signal fluctuation-to-noise ratio, and per-slice variation (Glover et al., 2012; https://www.nitrc.org/projects/bxh_xcede_tools/). Preprocessing analyses were carried out using the CONN functional connectivity toolbox (22 v.2407) in MATLAB (Whitfield-Gabrieli & Nieto-Castanon, 2012). Preprocessing steps included functional realignment and unwarping to estimate and correct for participant motion, distortion correction based on the field map, and a slice-timing correction that corrected for maturation of the BOLD signal over time (Huettel et al., 2004). Additionally, functional outliers were detected with an ART (Artifact Detection Tools)-based identification method (NITRIC, 2015), in which outliers were defined using a conservative threshold (i.e., 97th percentile) and subsequently removed. All anatomical and functional images were normalized into standard Montreal Neurological Institute (MNI) space. The anatomical images were segmented into gray matter, white matter, and cerebral spinal fluid (CSF) tissue classes with CONN, which uses Statistical Parametric Mapping's unified segmentation and normalization procedure. These masks were then applied to the functional images (Ashburner & Friston, 2005). During registration, functional images were aligned to anatomical images, and both were normalized to standard space. A smoothing kernel of 6 mm was used to increase the signal-to-noise ratio, as well as to reduce spurious activations of single voxels. During denoising, the representative noise signal from white matter (five components) and CSF (five components) was extracted, and any signal correlated with these components was removed from the BOLD signal. The noise removal used the CompCor approach, which extracts multiple signals from CSF and white matter areas to capture motion and physiological artifacts while excluding neural signals, which avoids introducing artifactual negative correlations in the connectivity measures (Chai et al., 2012; Liu et al., 2017). To eliminate frequencies of less interest, a band-pass filter (0.008 and 0.09 Hz) was used (Davey et al., 2013; Gohel & Biswal, 2015; Hallquist et al., 2013). The effects of the following quality assurance parameters were controlled for during data analysis: number of outlier and nonoutlier scans (outlier threshold = 0.5 mm), max and mean motion, and max and mean global BOLD signal changes (outlier threshold = global signal *z* value of 3). The average number of invalid scans was 1.70 out of 180 scans/volumes (0.9%, *SD* = 3.76), and it was significantly affected by age (*β* = 0.028, *SE* = 0.012, *p* = 0.041). The mean amount of motion was 0.192 mm (*SD* = 0.074 mm), and increased age was associated with higher head motion (*β* = 0.0015, *SE* = 0.00025, *p* < 0.001). The analyses removing variance associated with the variables described above occurred in a single linear regression step, and the residualized BOLD signal was used for further statistical analyses.

### Node Definition and Network Measures

Regions of interest (ROIs) were defined from the Power et al. (2011) parcellation, which comprises 264 coordinates. We selected a subset of these nodes corresponding to the DMN and MD networks and constructed 6-mm-radius spherical ROIs centered on the MNI coordinates. The language network was defined based on Fedorenko et al. (2010). We selected a set of left-lateralized ROIs from this atlas, including key regions in the inferior frontal and posterior temporal cortex (see Table 2 for details). All ROIs were constructed in MNI space and visually inspected in FSLeyes to confirm anatomical accuracy and lack of spatial overlap (McCarthy, 2025). Functional time series were extracted using the CONN Toolbox v22 (Whitfield-Gabrieli & Nieto-Castanon, 2012), and ROI-to-ROI connectivity values were used in all statistical models.

**Table 2.** Montreal Neurological Institute coordinates for default mode, language, and multiple demand network regions of interest

Region	*X*	*Y*	*Z*
Default mode network			
Left dorsal medial prefrontal cortex	−27	23	48
Right dorsal medial prefrontal cortex	27	23	48
Right posterior parietal cortex	41	−60	29
Left superior temporal sulcus	−64	−20	−9
Right superior temporal sulcus	64	−20	−9
Left ventromedial prefrontal cortex	−7	49	18
Right ventromedial prefrontal cortex	7	49	18
Left hippocampal formation	−25	−32	−18
Right hippocampal formation	25	−32	−18
Left posterior cingulate cortex	−7	−52	26
Right posterior cingulate cortex	7	−52	26
Language network			
Left temporal pole	−52	6	−18
Left anterior middle temporal gyrus	−54	−14	−14
Left inferior frontal gyrus, pars triangularis	−48	28	−4
Left posterior middle temporal gyrus	−56	−38	0
Left angular gyrus	−52	−54	12
Left inferior frontal gyrus, pars opercularis	−52	24	14
Left lateral occipital cortex	−42	−68	24
Left precentral gyrus	−44	−80	50
Multiple demand network			
Left posterior parietal cortex	−18	−66	52
Right posterior parietal cortex	18	−66	52
Left mid-parietal cortex	−44	−56	52
Right mid-parietal cortex	44	−56	52
Left anterior parietal cortex	−48	−38	48
Right anterior parietal cortex	48	−38	48
Left superior frontal gyrus	−30	0	58
Right superior frontal gyrus	30	0	58
Left precentral gyrus A	−48	6	38
Right precentral gyrus A	48	6	38
Left precentral gyrus B	−50	12	22
Right precentral gyrus B	50	12	22
Left middle frontal gyrus	−42	30	30
Right middle frontal gyrus	42	30	30
Left orbital middle frontal gyrus	−34	54	12
Right orbital middle frontal gyrus	34	54	12
Left insula	−34	22	−2
Right insula	34	22	−2
Left medial frontal gyrus	−8	20	42
Right medial frontal gyrus	8	20	42

We examined three RSFC metrics for each network: within-network connectivity, between-network connectivity, and segregation. Prior to averaging, self-correlations (diagonal elements) were excluded, and any negative correlations were set to zero due to uncertainty regarding their interpretation (Hallquist & Hillary, 2018). All remaining Pearson correlation coefficients were Fisher *z*-transformed to stabilize variance.

Within-network connectivity was calculated as the mean *z*-transformed correlation among all unique ROI pairs within a network, incorporating bilateral nodes for the DMN and MD but restricted to left-hemisphere ROIs for the language network. Between-network connectivity was defined as the mean *z*-transformed correlation between every ROI in one network and every ROI in a second network; we specifically evaluated connectivity between the language and DMN networks and the language and MD networks. Network segregation, which indexes the degree of functional differentiation, was computed following Chan et al. (2014) and Zhang and Diaz (2023) as the difference between within-network functional connectivity (FC) and between-network functional connectivity divided by within-network functional connectivity.

$$\text{Segregation}=\frac{\text{Within}-\text{network connectivity}-\text{Between}-\text{network connectivity}}{\text{Within}-\text{network connectivity}}$$

Higher segregation values indicate stronger cohesion within a network relative to its connectivity with other networks. For the language network, segregation reflects the distinction of left-lateralized language ROIs from both the DMN and MD, whereas for the DMN and MD networks, segregation quantifies each network’s specialization relative to the other rather than relative to the language network. See equations below for the exact equations.

$$BNC_{\text{Lang}}=\text{mean}\left({FC}_{\text{Lang}-DMN},{FC}_{\text{Lang}-MD}\;\right)$$

$$BNC_{DMN}={FC}_{DMN-MD}$$

$$BNC_{MD}={FC}_{MD-DMN}$$

This asymmetrical construction was intentional as we consider the DMN and MD as closely interacting domain-general networks whose reciprocal connectivity is often used to characterize age-related changes in functional organization, whereas the language network serves a more specialized language role. Including language connectivity in the denominator of DMN or MD segregation would conflate specialized vs. domain-general interactions. We argue here that our approach preserves the interpretive specificity of segregation for each network.

### Data Analyses

All data analyses were conducted in R (Version 4.5.0; R Core Team, 2025), using tidyverse (Wickham et al., 2019), broom (Robinson, 2014), lavaan (Rosseel, 2012), and mediation packages (Tingley et al., 2014). Behavioral and RSFC data from the three projects were combined. For two of the three project data sets, disfluency metrics from the two elicitation tasks (picture book and open-ended prompt) were averaged to yield a single measure per participant.

Age, Stroop interference, and RSFC predictors were mean centered to improve interpretability and reduce multicollinearity, particularly in interaction terms involving age and EF. Disfluency outcomes—total disfluencies, filled pauses, unfilled pauses, repetitions, revisions, and prolongations—were operationalized as the percentage relative to the total number of words produced, i.e., these values reflected the proportional frequency of each disfluency subtype in the speech stream rather than raw counts. Prior to statistical analyses, we inspected the distributions of all key variables using descriptive statistics (mean, median, range, skewness) generated with the describe function from the psych package in R (Revelle, 2026). Age and education were approximately normally distributed, whereas Stroop interference scores and disfluency measures showed the expected mild positive skew typical of reaction time and proportion data. No extreme outliers or data entry errors were detected. In addition, given the potential effect of educational attainment on cognition and (e.g., Braveman & Gottlieb, 2014; Chan et al., 2018; Hackman et al., 2010), education (in years) was included as a covariate in all analyses. Project was also added as a covariate to account for cohort-level differences in sampling and task implementation. All models were initially run for each disfluency subtype to evaluate associations with RSFC and EF. However, because our primary goal was to examine age-related variability in speech production, follow-up mediation analyses focused only on the outcomes that showed significant associations with age and/or EF, i.e., total disfluencies, revisions, and filled pauses (see details in Results).

### Predicting Disfluency From Age, EF, and Network Segregation

To evaluate whether age, EF, and network segregation independently or interactively predicted disfluency production, we ran separate linear models for each disfluency type. The model specifications followed this base structure: Disfluency Outcome ∼ Age + Stroop + Network Segregation + Age × Stroop + Age × Network Segregation + Education + Project. These models were run once for each of the three networks (language, MD, DMN), yielding 3 networks × 6 outcomes for 18 models total. Note that separate models for within- and between-network connectivity (rather than segregation) were also run; these results are reported in the Supplementary Materials (Supporting Information can be found at https://doi.org/10.1162/NOL.a.245). Raw *p* values for all segregation main effects and all Age × Segregation interaction tests were each false discovery rate-adjusted (Benjamini & Hochberg, 1995), with *q* < 0.05 taken as significant. Significant interaction terms were probed using simple slopes and Johnson–Neyman tests. In what follows, we report unstandardized coefficients, 95% confidence intervals, and *p* values, focusing on effect size interpretation in line with recommendations for estimation-based reporting (Cumming, 2014).

### Mediation Analyses

To examine whether the significant relationships between age and disfluency production were mediated by EF or network segregation, we conducted parallel mediation analyses focused on the four disfluency outcomes that showed a significant age effect (repetitions, revisions, filled pauses, unfilled pauses). For each mediator (1) Stroop interference and (2) segregation in each network (language, MD, and DMN), we estimated three paths: the effect of age on the mediator (path a), the effect of the mediator on disfluency (path b), and the effect of age on disfluency with and without the effect of the mediator (paths c and c′). Significant indirect effects were determined using nonparametric bootstrapping with 5,000 resamples. Although model fit indices were returned by default, we evaluated models based on path estimates and bootstrapped confidence intervals, rather than global fit (Hayes, 2017).

Lastly, we considered more complex serial models to test whether network segregation impacts speech via EF (i.e., age → RSFC → EF → disfluency); our results, however, did not support key prerequisite paths for this approach. For example, DMN segregation did not significantly predict EF performance, precluding formal serial mediation. Thus, our main focus remained on independent mediation pathways for EF and network segregation.

## RESULTS

### Main Effects of Age on EF, Production, and Network Segregation

To isolate the effect of age, we first conducted separate linear regression models predicting the effect of age on Stroop interference, disfluency subtypes, and network segregation, controlling for education and project. These simplified models provide clean estimates of age-related differences and are reported below independently of the full models that incorporate network segregation and EF as additional predictors.

Analyses revealed a significant effect of age on Stroop interference scores (*b* = 1.72, *SE* = 0.27, *p* < 0.001, 95% CI [1.19, 2.24]) such that as age increased, Stroop interference increased, i.e., older age was associated with worse EF. For disfluency production, analyses revealed that older age was associated with significantly more repetitions, *b* = 0.006, *SE* = 0.003, *t* = 2.18, *p* = 0.030, 95% CI [0.001, 0.012], *q* = 0.045; more unfilled (silent) pauses, *b* = 0.017, *SE* = 0.007, *t* = 2.37, *p* = 0.018, 95% CI [0.003, 0.031], *q* = 0.037; more revisions, *b* = 0.006, *SE* = 0.002, *t* = 2.62, *p* = 0.009, 95% CI [0.001, 0.010], *q* = 0.028; and fewer filled pauses, *b* = −0.025, *SE* = 0.008, *t* = −3.26, *p* < 0.001, 95% CI [−0.040, −0.010], *q* = 0.008. The age effect of fewer filled pauses with increasing age was due to lexical fillers (e.g., “you know,” “like”) of which younger adults produced more. Age was not associated with overall disfluency production, *b* = 0.002, *SE* = 0.017, *t* = 0.11, *p* = 0.916, 95% CI [−0.031, 0.035], *q* = 0.916, nor with prolongations, *b* = −0.010, *SE* = 0.008, *t* = −1.23, *p* = 0.219, 95% CI [−0.026, 0.006], *q* = 0.263.

For network segregation, analyses revealed that older age was significantly associated with declines across all three systems: Language network segregation decreased (*b* = −0.002, *SE* = 0.001, *t* = −2.52, *p* = 0.012, 95% CI [–0.003, 0.000], *q* = 0.012), DMN segregation decreased (*b* = −0.002, *SE* = 0.001, *t* = −3.07, *p* = 0.002, 95% CI [−0.004, –0.001], *q* = 0.007), and MD network segregation decreased (*b* = −0.002, *SE* = 0.001, *t* = −2.78, *p* = 0.006, 95% CI [−0.004, −0.001], *q* = 0.009). These results suggest that older age is associated with reduced functional specialization within key networks supporting language and domain-general cognition (results for within- and between-network connectivity are reported in the Supplementary Materials).

### Network Segregation Predicts Disfluency

Next, we examined whether resting-state network segregation predicted speech disfluency outcomes and whether these effects were moderated by age or EF. Separate linear models were conducted for each metric across each network and for each disfluency subtype.

Only DMN segregation emerged as a significant predictor of speech disfluency patterns. Greater DMN segregation was significantly associated with fewer total disfluencies (*b* = −3.43, *SE* = 1.47, *t* = −2.34, *p* = 0.020, 95% CI [−6.32, −0.54], *q* = 0.041), fewer repetitions (*b* = −0.63, *SE* = 0.25, *t* = −2.56, *p* = 0.011, 95% CI [−1.12, −0.15], *q* = 0.033), and fewer revisions (*b* = −0.59, *SE* = 0.19, *t* = −3.06, *p* = 0.002, 95% CI [−0.97, −0.21], *q* = 0.015).

A significant interaction between age and DMN segregation was observed for repetitions (*b* = 0.042, *SE* = 0.014, *t* = 2.91, *p* = 0.004, 95% CI [0.014, 0.070], *q* = 0.024), indicating that the relationship between segregation and repetition disfluencies varied with age. Follow-up simple slopes analyses showed that greater DMN segregation predicted significantly fewer repetitions at younger ages (*b* = −1.35, *SE* = 0.39, *t* = −3.46, *p* = 0.001), a weaker but still significant effect at middle age (*b* = −0.64, *SE* = 0.25, *t* = −2.57, *p* = 0.011), and no effect for older adults (*b* = 0.08, *SE* = 0.30, *t* = 0.27, *p* = 0.79). The Johnson–Neyman analysis further confirmed that the effect of DMN segregation on repetitions was statistically significant (*p* < 0.05) for individuals younger than age ~47.6 years. These results align with previous results demonstrating that whole-brain network segregation is more strongly associated with better language production in younger and middle-aged adults, but not older adults (Zhang & Diaz, 2023).

In contrast, MD segregation and language network segregation were not significantly associated with any disfluency subtype, and no age interactions emerged. Despite our hypotheses about their role in executive control and speech production, neither network's segregation metric contributed to predicting individual differences in disfluency once age and Stroop performance were accounted for.

In summary, these results highlight that higher DMN segregation, more than language and MD network segregation, predicts lower disfluency. Age-related decreases in DMN segregation therefore may be one neural mechanism underlying speech fluency errors with aging. We discuss this in detail in the Discussion.

### Mediation Analyses

**Figure 1.**
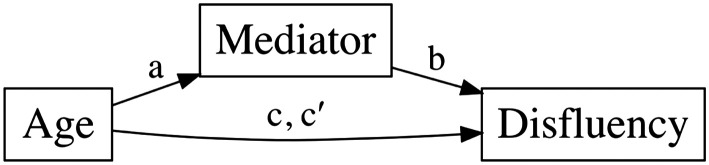
Conceptual mediation framework. Conceptual mediation framework used to test whether executive function (EF) or resting-state functional network segregation mediates the relationship between age and speech disfluency. The total effect of age on disfluency is represented by path c. The mediation model decomposes this effect into an indirect pathway (Age → Mediator → Disfluency; paths a and b) and a direct effect (c′), controlling for the mediator.

We next conducted a series of mediation analyses to test whether EF and/or resting-state functional network segregation mediated the relationship between age and speech disfluency. Based on prior linear modeling results, we focused only on DMN segregation and disfluency types that showed significant age effects, specifically repetitions, revisions, unfilled pauses, and filled pauses. Figure 1 illustrates a conceptual mediation framework.

### EF as a Mediator of Age–Disfluency Relationships

We first examined whether EF (the Stroop effect) mediated the relationship between age and revisions. Analyses revealed no evidence that EF mediated the relationship between age and revisions. Although age significantly predicted both worse Stroop performance (*p* < 0.001) and more revisions (*p* = 0.009), Stroop did not significantly predict revisions (*p* = 0.78). Consistent with this pattern, the estimated indirect effect was not significant (Average Causal Mediated Effect [*ACME*] = 0.00025, 95% CI [−0.0015, 0.00], *p* = 0.78), but the direct effect of age remained significant (Average Direct Effect [*ADE*] = 0.00554, *p* = 0.024), indicating no mediation by Stroop performance. Similarly, the Stroop effect did not significantly predict filled pauses (*p* = 0.30). The indirect effect of Stroop was not significant (*ACME* = −0.00326, 95% CI [−0.00840, 0.000], *p* = 0.21), while the direct effect of age on filled pauses remained significant (*ADE* = −0.0215, *p* = 0.023), again indicating no mediation. Repetitions were also not mediated by EF with an indirect effect of (*ACME* = −0.00326, *p* = 0.21). Lastly for unfilled pauses, again EF did not significantly predict unfilled pauses (*p* = 0.96), with the estimated indirect effect of (*ACME* = 0.00016, 95% CI [−0.0048, 0.01], *p* = 0.92) and direct effect of age (*ADE* = 0.01688, *p* = 0.028). In summary, while EF declined with age overall, it did not relate to the age-related increase in speech repetitions, revisions, unfilled pauses, or the decrease in filled pauses.

### DMN Segregation as a Mediator of Age–Disfluency Relationships

Next, we examined whether RSFC measures mediated the relationship between age and speech disfluencies. Analyses revealed mediation effects only for revisions such that two RSFC pathways showed evidence of partial mediation. First, older age was associated with reduced DMN segregation (*b* = −0.0023, *p* = 0.002), and lower DMN segregation predicted more revisions (*b* = −0.59, *p* = 0.002). Mediation analysis confirmed a significant indirect effect (*ACME* = 0.0012, 95% CI [0.0002, 0.0040], *p* = 0.012). The direct effect of age on revisions remained significant (ADE = 0.0046, *p* = 0.038), indicating partial mediation. Approximately 20.6% of the total effect of age on revisions was mediated by DMN segregation (*p* = 0.020).

**Figure 2.**
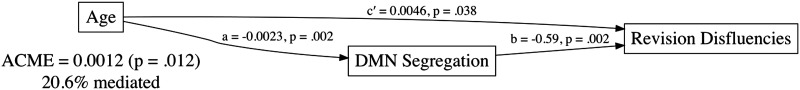
Default mode network (DMN) mediation for revisions. Significant mediation model showing that DMN segregation partially mediates the association between age and revision disfluencies. Older age was associated with lower DMN segregation (path a), and lower DMN segregation predicted more revisions (path b).

Finally, we tested whether network segregation influenced speech disfluency through its effect on EF (i.e., Age → Segregation → Stroop → Disfluency). In both models, segregation did not significantly predict Stroop performance. The indirect effect of age on disfluency via Stroop was not significant (*ACME* = 0.00427, 95% CI [−0.0121, 0.02], *p* = 0.62), and the direct effect of age (*ADE* = −0.00839, 95% CI [−0.0435, 0.03], *p* = 0.67) was nearly equal to the total effect (−0.00412), suggesting no evidence of serial mediation. See Figure 2 for a schematic of model mediation of age effects on revision disfluencies by DMN segregation.

## DISCUSSION

This study examined how age, EF (operationalized as Stroop performance), and RSFC relate to naturalistic speech disfluencies in adults across the lifespan. Our findings reveal that increased age is associated with increases in specific disfluency types, namely, repetitions, revisions, and unfilled pauses, and fewer filled pauses. Notably, DMN segregation predicted fewer overall disfluencies, repetitions, revisions, irrespective of age, and partially mediated the relationship between age and revision disfluencies, suggesting that age-related changes in large-scale network organization may contribute to planning and/or monitoring related speech disruptions.

In contrast, although age was associated with poorer EF, i.e., poorer Stroop performance, Stroop interference did not predict any disfluency subtype patterns, nor did it mediate the link between age and disfluency by subtype. Together, these findings suggest that fluent speech may rely not only on language-specific resources but also on domain-general neural organization, especially within the DMN, and that age-related disruption to this system may contribute to distinct patterns of age-related speech disfluency.

### The Role of Functional Segregation in Speech Fluency

Our findings suggest that functional segregation within the DMN plays a central role in supporting fluent speech. Greater DMN segregation was associated with fewer disfluencies overall, especially fewer repetitions and revisions. In contrast, segregation within the language and MD networks did not predict disfluencies by type or collectively. These findings highlight the unique contribution of domain-general organization in spontaneous speech. There was also a significant effect of age on the relationship between DMN segregation and repetitions such that this relationship was strongest among younger and middle-aged adults. This finding is consistent with weaker brain–behavior relationships among older adults. Moreover, DMN segregation partially mediated the relationship between age and revisions, suggesting that age-related declines in domain-general network specialization may contribute to difficulties monitoring speech. Collectively, these findings suggest that DMN segregation is an important factor in reducing speech disfluencies. It is important to reiterate how segregation was defined in the present study, as our measure reflects differentiation among the three a priori networks (language, DMN, MD) rather than whole-brain segregation. For the language network, between-network connectivity was calculated as its mean connectivity with both the DMN and MD, whereas for the DMN and MD, between-network connectivity reflected their reciprocal DMN–MD coupling. Accordingly, the DMN segregation effects reported here should be interpreted as reflecting the distinctiveness of the DMN specifically from the MD network, rather than global network specialization. Thus, while these findings can be framed as reduced disfluencies, they also highlight that stronger DMN-MD segregation may actively facilitate more efficient, well-coordinated speech processes, but potentially through distinct mechanisms for revisions (monitoring/correction) versus repetitions (planning/coordination).

These results align with recent work by Gordon et al. (2020), highlighting the DMN’s integrative function across cognitive domains, which proposes that the DMN contains distinct streams that couple selectively with either the language system or the executive control network. Such streams may support the integration of conceptual and linguistic processing with goal-directed behavior. Thus, reduced segregation within the DMN, as seen with aging in the present study, may reflect weakened differentiation between these streams, potentially increasing interference between self-referential, linguistic, and executive processes during speech.

This interpretation is also consistent with neural dedifferentiation accounts, which propose age-related reductions in network specificity (Chan et al., 2014; Goh, 2011; Li et al., 2001). In our data, DMN segregation no longer predicted disfluency in the oldest participants, suggesting that the protective effects of segregation are not uniformly maintained across the lifespan. Zhang and Diaz (2023) similarly reported that although segregation within the language network was stable with age, whole-brain network segregation declined in older adults and more strongly predicted language production ability than language network segregation alone. Furthermore, their moderation analyses revealed that the benefits of segregation for language production were largely confined to younger and middle-aged adults—paralleling our finding that DMN segregation was most predictive of disfluency in earlier adulthood. While we did see age-related reductions in network segregation for both the language and MD networks, neither of these related to disfluencies. Together, these results suggest that broader, domain-general network organization—not language-specific connectivity per se—may play a critical role in supporting fluent language production across the lifespan.

In addition, the absence of age-related effects on language network segregation is theoretically meaningful. Rather than indicating insensitivity of our speech tasks or the need for more difficult paradigms, this pattern is consistent with a substantial body of evidence demonstrating the resilience of the language network across the lifespan (e.g., Campbell et al., 2016; Diaz et al., 2021, 2022; Zhang & Diaz, 2023). Prior work has shown that core frontotemporal language regions maintain stable functional specialization and relatively preserved connectivity even in the context of broader age-related declines in domain-general networks. In other words, the relative stability of language network segregation we observed here suggests that age-related increases in disfluency are unlikely to reflect degradation of the language network itself. Instead, our findings support the interpretation that disfluency patterns in aging arise from changes to domain-general systems, most notably the DMN, rather than from declines within the language network.

A further consideration is that spontaneous speech disfluencies do not arise from variation within the language network itself. Contemporary models largely agree on the view that the frontotemporal language system supports linguistic representation, i.e., lexical access/retrieval, semantic integration, and syntactic binding, but that it does not directly implement the higher-order cognitive processes that support language processing and production (e.g., Fedorenko & Thompson-Schill, 2014; Hagoort, 2013; Matchin & Hickok, 2020). In particular, Fedorenko and Thompson-Schill (2014) argue that the language network comprises a specialized core that selectively supports linguistic computations, whereas a domain-general periphery (including the DMN and MD network) is flexibly recruited when tasks require monitoring, conflict resolution, or additional processing effort. This distinction aligns with our interpretation that disfluencies likely reflect failures in domain-general coordination rather than impairments in representational linguistic processes. Although our preregistered hypotheses included the language network as a candidate system that might predict fluency, the present findings are consistent with the broader theoretical framework, suggesting that disfluencies may arise from demands on conceptual planning, internal mentation, error monitoring, and self-initiated adjustments—processes supported by domain-general networks, particularly the DMN.

Our findings also add clarity to the mechanisms linking aging and disfluency. Specifically, DMN segregation partially mediated the relationship between age and revision disfluencies, suggesting that age-related declines in functional specialization may undermine the cognitive scaffolding needed for fluent speech. In younger and middle-aged adults, greater segregation may protect against disfluency by limiting interference from competing internal processes and supporting efficient coordination between conceptual and linguistic operations. In contrast, diminished segregation in older adults may reflect a breakdown in this modular architecture, reducing the brain’s ability to flexibly manage self-monitoring demands during speech. This pattern is consistent with models of functional reorganization in aging (e.g., Cabeza et al., 2002; Spreng & Turner, 2019), which suggest that the aging brain adapts by reallocating cognitive resources, albeit with trade-offs in efficiency. In the domain of language production, such reorganization may come at the cost of fluency, as increased reliance on more interconnected neural systems may hinder the rapid planning, monitoring, and correction required for fluent speech.

### Clarifying Mechanisms Across Disfluency Subtypes

To further examine how aging may influence speech fluency, we tested whether EF or RSFC mediated the relationship between age and specific disfluency types. While age was significantly associated with worse EF performance, EF did not significantly predict disfluency subtypes and did not mediate any age–disfluency relationships. Because the Stroop effect primarily indexes inhibitory control under rapid response selection, rather than EF globally, this null finding suggests that the specific inhibitory demands captured by this task may potentially play a limited role in supporting fluent spontaneous speech. This interpretation is consistent with more recent work showing limited associations between EF and disfluency in older adults (Beier et al., 2023; Engelhardt & Markostamou, 2025).

Although some studies have linked EF (encompassing inhibition and working memory) to specific disfluency types (e.g., repetitions and filled pauses), these effects have been shown to be dependent on task demands and may not generalize to spontaneous speech (e.g., Engelhardt & Markostamou, 2025). Therefore, the absence of EF–disfluency associations in our data does not necessarily imply that EF does not play a role in speech production. Rather, it underscores that different EF subcomponents are likely engaged depending on the linguistic and cognitive demands of the task.

In contrast, RSFC patterns, specifically reduced DMN segregation, partially explained age-related increases in revision disfluencies. These findings suggest that intrinsic neural organization, rather than cognitive control performance, may be a key contributor to age-related changes in speech fluency, especially for disfluencies that involve mid-sentence corrections.

Moreover, the mediation analyses results suggest that speech disfluency subtypes may arise from distinct neural mechanisms. While EF was not a significant mediator, disruptions to DMN organization appears to be a potential pathway linking age to increased revisions in speech production. These results also extend prior work on age and disfluency (e.g., Beier et al., 2023; Engelhardt & Markostamou, 2025) by identifying neural-level mediators that may underlie fluency decline in the absence of behavioral predictors like EF.

Importantly, these brain–behavior relationships differed by disfluency subtype. RSFC segregation varied across subtypes, highlighting the need to treat disfluency as a heterogeneous construct. No disfluencies were linked to EF, whereas total disfluencies, repetitions, and revisions showed stronger associations with RSFC. Certain speech disfluencies may therefore reflect distinct mechanisms, including monitoring demands, planning effort, and network coordination.

### Limitations and Future Directions

While this study offers new insights into the network-level predictors of disfluency across adulthood, several limitations should be considered. First, because we focused naturalistic speech elicitation, we did not manipulate task difficulty or include experimental manipulations that could clarify the causal relationship between cognitive control and speech disfluency. The absence of an EF–language relationship in this context may initially seem surprising, given prior work linking EF to aspects of sentence processing. However, this may reflect the nature of the task itself such that spontaneous, narrative discourse likely places relatively modest demands on executive control compared to paradigms that require processing, for example, syntactically complex sentences. Prior studies that find a stronger EF–language relationship often use such demanding experimental designs, whereas the present study examined unconstrained, naturalistic speech where EF may play a less central role.

Second, although we quantified disfluency production using proportional metrics (e.g., percentage of filled pauses), we did not examine how disfluencies impact global or local coherence in discourse, e.g., whether a revision clarified the message. Moreover, we did not examine the effect of disfluencies on the listener, as the speech elicitation tasks were essentially monologues.

Disfluencies can vary in how disruptive or functional they are to the listener, depending on their type and placement within a narrative. For instance, filled pauses may serve as markers of planning, while repetitions or revisions could signal breakdowns that affect how well the listener follows the speaker's intended message. Future work should assess not only the occurrence or frequency of disfluencies but also their consequences for global coherence (i.e., thematic consistency across a discourse) and local coherence (i.e., logical flow between adjacent utterances). Integrating this type of linguistic analyses of coherence with RSFC and cognitive measures could provide a more nuanced understanding of how neural organization supports meaningful, effective communication in aging.

### Conclusion

This study provides new insight into how aging affects speech fluency, revealing that RSFC, particularly the functional organization of the DMN, plays an important role in impacting disfluencies across adulthood. By differentiating disfluency subtypes, we identified specific links between DMN segregation and fewer total disfluencies, repetitions, and revisions, highlighting the importance of domain-general network integrity in fluent speech production. In contrast, EF did not reliably predict disfluencies, and RSFC did not mediate EF–speech relationships, suggesting that intrinsic brain organization may contribute to speech fluency independently of cognitive control. These findings underscore the utility of RSFC as a tool for understanding how large-scale brain network architecture supports speech production and how these systems shift with age. In summary, fluent speech depends not only on the integrity of the language network but also on the broader differentiation of intrinsic networks such as the DMN. Examining RSFC alongside behavioral measures offers a powerful lens for understanding how communication changes over the adult lifespan.

## Funding Information

Michele T. Diaz, National Institute on Aging (https://dx.doi.org/10.13039/100000049), Award ID: R01 AG034138. Megan S. Nakamura was supported by, National Institute on Aging (https://dx.doi.org/10.13039/100000049), Award ID: T32 AG049676. Haoyun Zhang, Science and Technology Development Fund, Macao S.A.R., Award ID: FDCT, 0153/2022/A.

## Author Contributions

**Megan S. Nakamura:** Conceptualization: Supporting; Formal analysis: Lead; Writing – original draft: Lead. **Haoyun Zhang:** Conceptualization: Equal; Formal analysis: Equal; Funding acquisition: Equal; Investigation: Equal; Writing – review & editing: Equal. **Michele T. Diaz:** Conceptualization: Equal; Data curation: Equal; Formal analysis: Supporting; Funding acquisition: Lead; Investigation: Equal; Project administration: Lead; Writing – review & editing: Equal.

## Data Availability Statement

The data and analysis scripts are openly available on the OSF at: https://osf.io/vp9za. Raw imaging data may be available upon request.

## Supplementary Material



## TECHNICAL TERMS

Resting-state functional connectivity (RSFC): The extent to which the activity in different brain regions is correlated during rest.
Default mode network (DMN): A set of regions, typically active when not actively engaged in an external task, that has been associated with internally directed cognition (e.g., autobiographical memory, mind wandering, planning, and introspection).
Multiple demand (MD) network: A set of regions in frontal and parietal cortices that supports domain-general higher cognitive functions including cognitive control, working memory, and attention among others. These regions are also referred to as the frontoparietal control network.
Network segregation: A measure of how distinct functional brain activation is between and within networks. It is calculated as the difference between within-network functional connectivity and between-network functional connectivity divided by within-network functional connectivity. Higher network segregation values indicate stronger cohesion within a network relative to its connectivity with other networks, and higher network segregation values have been associated with better cognitive performance.
