# Supplementary material for: Age-Related Differences in Resting-State Functional Connectivity Predict Specific Patterns of Speech Disfluency
Source: Neurobiol Lang (Camb). 2026 Apr 23;7:NOL.a.245. doi: 10.1162/NOL.a.245 (PMC13137885; doi:10.1162/NOL.a.245)
Supplement: Supplementary file 1 [file nol-07-245-s001.pdf]

**Supplemental Materials**

1. Age Effects on Network Connectivity

Age was significantly associated with declines in both within- and between-network resting-state functional connectivity (RSFC). Within-network connectivity decreased significantly with age across all three networks of interest. Specifically, connectivity declined in the Language network ($b$ = –0.001, $SE$ = 0.000, $t$ = –2.759, $p$ = .006, 95% CI [–0.002, 0.000], $q$ = .010), the Default Mode Network (DMN; $b$ = –0.002, $SE$ = 0.000, $t$ = –3.233, $p$ = .001, 95% CI [–0.002, –0.001], $q$ = .006), and the Multiple Demand (MD) network ($b$ = –0.001, $SE$ = 0.000, $t$ = –3.847, $p$ < .001, 95% CI [–0.002, 0.000], $q$ = .001). For between-network metrics, older age was significantly associated with reduced functional coupling between the Language and DMN networks ($b$ = –0.001, $SE$ = 0.000, $t$ = –2.439, $p$ = .015, 95% CI [–0.001, 0.000], $q$ = .018). In contrast, connectivity between the Language and MD networks was not significantly related to age ($b$ = 0.000, $SE$ = 0.000, $t$ = 1.251, $p$ = .212, $q$ = .212).

2. Connectivity Predictors of Disfluency

Among within-network connectivity metrics, only DMN connectivity significantly predicted speech disfluency after correction for multiple comparisons. Greater within-DMN connectivity was associated with fewer revision disfluencies ($b$ = –0.85, $SE$ = 0.30, $t$ = –2.83, $p$ = .005, 95% CI [–1.45, –0.26], $q$ = .030), whereas no other disfluency subtype showed a significant relationship with within-network DMN connectivity.

In the case of between-network metrics, stronger connectivity between the Language and DMN networks was also associated with fewer revision disfluencies ($b$ = –1.40, $SE$ = 0.49, $t$ = –

2.89, *p* = .004, 95% CI [–2.36, –0.45], *q* = .025). Although increased between-network connectivity is often interpreted as a marker of dedifferentiation or reduced functional specialization (e.g., (Chan et al., 2014; Li et al., 2001; Zhang et al., 2021), these findings suggest that in the context of spontaneous speech production, stronger Language–DMN coupling may specifically support more efficient speech planning and output.

3. Mediation Analysis: Language–DMN Connectivity

Exploratory mediation analyses examined whether Language–DMN connectivity mediated the relationship between age and revision disfluencies. Consistent with the correlational findings, age significantly predicted lower Language–DMN connectivity (*b* = –0.00072, *p* = .016), and lower connectivity in turn predicted a greater number of revisions (*b* = –1.40, *p* = .004). The indirect effect of age on revisions via Language–DMN connectivity was marginally significant, with an average causal mediation effect (ACME) of 0.00085 (95% CI [–0.000030, 0.000], *p* = .060). The direct effect of age on revisions remained significant (*ADE* = 0.0054, *p* = .015), suggesting that Language–DMN connectivity accounts for a small, indirect portion of the age-related increase in revision disfluencies. Approximately 13.6% of the total age effect was mediated through Language–DMN connectivity (*p* = .11), although this proportion did not reach conventional levels of significance.
